# Epidemiology of HIV in pregnant women and its relationship with the period of the COVID-19 pandemic

**DOI:** 10.1590/1980-220X-REEUSP-2022-0339en

**Published:** 2023-03-27

**Authors:** Bárbara Brandão Lopes, Ane Kelly Lima Ramalho, Mônica Oliveira Batista Oriá, Gilmara Holanda da Cunha, Priscila de Souza Aquino, Ana Karina Bezerra Pinheiro

**Affiliations:** 1 Universidade Federal do Ceará, Departamento de Enfermagem, Programa de Pós-graduação em Enfermagem, Fortaleza, CE, Brazil.

**Keywords:** HI, Pregnanc, Health Promotio, Epidemiolog, COVID-19, VIH, Embarazo, Promoción de la Salud, Epidemiología, COVID-19, HIV, Gravidez, Promoção da Saúde, Epidemiologia, COVID-19

## Abstract

**Objective:**

To analyze, in the light of the Social Ecological Theory, the progression of reported cases of HIV during pregnancy in a Brazilian state and their relationship with the onset of the COVID-19 pandemic.

**Method:**

Retrospective study, with a sample consisting of all reports of gestational HIV in the state of Ceará - Brazil from 2017 to 2021, on the IntegraSUS platform. Data collection was carried out in January 2022. The analyzed variables were organized according to the theoretical levels: macrosystem, exosystem, mesosystem, and microsystem.

**Results:**

A total of 1,173 cases of HIV in pregnant women were recorded. When comparing the pre- and post-pandemic period, a reduction in the disease detection rate (from 231 to 122.67 pregnant women) was observed, as well as 1.82 times more chances of women not using antiretrovirals during childbirth after the start of the pandemic. There was a 55% reduction in vaginal births and 39% in cesarean sections among women diagnosed with HIV after the start of the pandemic.

**Conclusion:**

The COVID-19 pandemic had an epidemiological and care impact, leading to a reduction in the number of notifications and in the detection rate of pregnant women living with HIV in the state of Ceará. Therefore, the need to ensure health care coverage is emphasized, with early diagnosis actions, guaranteed treatment, and quality prenatal care.

## RESUMO

**Objetivo:**

Analisar, à luz da Teoria Social Ecológica, a evolução dos casos notificados de HIV na gestação em um estado brasileiro e sua relação com o início da pandemia de COVID-19.

**Método:**

Estudo retrospectivo, com amostra composta por todas as notificações de HIV gestacional do estado do Ceará - Brasil no período de 2017 a 2021, na plataforma IntegraSUS. A coleta de dados foi realizada em janeiro de 2022. As variáveis analisadas foram organizadas de acordo com os níveis teóricos: macrossistema, exossistema, mesossistema e microssistema.

**Resultados:**

Foram registrados 1.173 casos de HIV em gestantes. Ao comparar o período pré e pós-pandemia, observou-se redução da taxa de detecção da doença (de 231 para 122,67 gestantes) e 1,82 vezes mais chances de as mulheres não usarem antirretrovirais no parto após o início da pandemia. Houve redução de 55% de partos vaginais e de 39% de cesarianas das mulheres com diagnóstico de HIV após o início da pandemia.

**Conclusão:**

A pandemia de COVID-19 causou impacto epidemiológico e assistencial, levando a uma redução do número de notificações e taxa de detecção de gestantes que vivem com HIV no estado do Ceará. Logo, reforça-se a necessidade de assegurar a cobertura de atenção à saúde, com ações de diagnóstico precoce, garantia de tratamento e uma assistência pré-natal de qualidade.

## RESUMEN

**Objetivo:**

Analizar, a la luz de la Teoría Ecológica Social, la evolución de los casos notificados de VIH durante el embarazo en un estado brasileño y su relación con el inicio de la pandemia de COVID-19.

**Método:**

Estudio retrospectivo, con una muestra compuesta por todos los informes de VIH gestacional en el estado de Ceará - Brasil de 2017 a 2021, en la plataforma IntegraSUS. La recolección de datos se realizó en enero de 2022. Las variables analizadas se organizaron según los niveles teóricos: macrosistema, exosistema, mesosistema y microsistema.

**Resultados:**

Se registraron 1.173 casos de VIH en gestantes. Al comparar el período pre y pospandemia, hubo una reducción en la tasa de detección de la enfermedad (de 231 a 122,67 mujeres embarazadas) y 1,82 veces más posibilidades de que las mujeres no usaran antirretrovirales durante el parto después del inicio de la pandemia. Hubo una reducción del 55% en los partos vaginales y del 39% en las cesáreas entre las mujeres diagnosticadas con VIH después del inicio de la pandemia.

**Conclusión:**

La pandemia de COVID-19 tuvo un impacto epidemiológico y asistencial, lo que provocó una reducción en el número de notificaciones y en la tasa de detección de mujeres embarazadas que viven con el VIH en el estado de Ceará. Por lo tanto, se refuerza la necesidad de asegurar la cobertura de salud, con acciones de diagnóstico temprano, tratamiento garantizado y atención prenatal de calidad.

## INTRODUCTION

Since 1980, it has been possible to observe a progressive increase in the number of AIDS cases among women, a period known as the feminization of the epidemic. This increase had repercussions on the rise in cases of women of childbearing age and, consequently, on the increase in vertical transmission^(1,2)^.

Knowledge of the serological status of the infection and the early diagnosis allowed the provision of adequate care for pregnant women with the Human Immunodeficiency Virus (HIV), with the implementation of public policies, in view of measures aimed at providing serological tests for viral detection during prenatal care and prophylactic treatment with antiretroviral therapy (HAART). Such policies had a positive impact, with changes in the profile of morbidity and mortality and a reduction in the risk of vertical transmission after the introduction of HAART^(3,4)^.

In Brazil, from 2000 to June 2021, 141,025 pregnant women with HIV were notified, of which 37.4% were residents of the Southeast region, followed by the South (29.5%), Northeast (18.3%), North (8.9%), and Midwest (5.9%). It was also observed that, from 2010 to 2020, there was a 30.3% increase in the HIV detection rate in pregnant women, which can be explained, in part, by the expansion of prenatal diagnosis and the improvement of surveillance in prevention of mother-to-child HIV transmission. Even in this context, the North and Northeast regions had the highest increases in this rate in the last 10 years, with 111.3% and 73.8%, respectively^(5)^.

In addition to HIV, there is the current confrontation with the Coronavirus-19 (COVID-19) pandemic. A systematic review and meta-analysis involving 42 studies and 438,548 pregnant women were carried out, which aimed to verify the impact of COVID-19 on maternal and fetal indicators. It was identified that when compared with pregnant women without infection, COVID-19 was associated with preeclampsia (OR 1.33, CI 95% 1.03 to 1.73), premature birth (OR 1.82, CI 95% 1.38 to 2.39), and stillbirth (OR 2.11, 95% CI 1.14 to 3.90). Compared with mild COVID-19, severe COVID-19 was strongly associated with preeclampsia (OR 4.16, 95% CI 1.55 to 11.15), premature birth (OR 4.29, 95% CI 2 .41 to 7.63), gestational diabetes (OR 1.99, 95% CI 1.09 to 3.64), and low birth weight (OR 1.89, 95% CI 1.14 to 3.12)^(6)^.

Aiming at providing comprehensive and quality care to mothers with HIV and protecting their babies, as well as promoting actions that minimize contamination rates, it is important to consider the socio-spatial realities in which these women are inserted^(7)^. It is also important to highlight that all three vaccines against COVID-19, two mRNA vaccines (from Pfizer-BioNTech, New York, NY and Moderna, Cambridge, MA) and an adenoviral vector vaccine (Johnson & Johnson–Janssen, Belgium), currently available in the United States, can be administered to pregnant or lactating women, without preference for the type of vaccine^(8)^.

In addition to the immunization action, the identification of the epidemiological profile of HIV in pregnant women allows understanding the social context and identifying the social determinants that relate women’s vulnerability to HIV infection. In the planning and elaboration of nursing strategies and actions, considering the provision of qualified prenatal care is of utmost importance, as it is an opportune moment for embracement, sensibilization, and construction of a bond of trust among the pregnant woman, her partner, and the team. In addition, knowing this profile can be seen as a valuable tool for assessing aspects related to sexual and reproductive health in search of better health promotion and disease prevention actions, contributing positively to the quality of care for women during pregnancy^(7,9,10)^.

Maternal and neonatal morbidity and mortality rates in Brazil are due to sociocultural inequalities within the regions of the country. A study aiming at describing the trend of neonatal mortality that is preventable by interventions of the Brazilian Public Health System, from 2000 to 2018, according to groups of causes of death and maternal residence, found a reduction in the rates of preventable neonatal mortality in all regions of Brazil. However, the North and Northeast regions had the highest rates of preventable neonatal mortality^(11)^.

Furthermore, regarding the maternal outcomes observed in the Northeast region of Brazil, a high rate of maternal mortality is observed, which can be interpreted by the socioeconomic vulnerability of the region, with higher poverty rates and lower coverage of care, compared to the South and Southeast Brazil^(12)^. Among the states in the Northeast region, Ceará is a federative unit with high socioeconomic vulnerability and social inequalities, since after the beginning of the pandemic, the poorest 50% of the population survive with an average monthly income of R$314 (less than US$ 60.00)^(13)^. In the context of the COVID-19 crisis, social and health care indicators were worse.

Based on the assumption of Social Ecological Theory (SET)^(14,15)^ in which the health and disease process is the result of the interaction of various social, political and economic elements, the use of the aforementioned theoretical framework was chosen for the conduction of the present study. The aim is to analyze the epidemiology of HIV in the pregnancy-puerperal period, comparing it before and after the onset of the pandemic.

Therefore, the objective was to analyze, in the light of the Social Ecological Theory, the progression of reported cases of HIV during pregnancy in a Brazilian state and their relationship with the onset of the COVID-19 pandemic.

## METHODS

### Design of Study

Retrospective descriptive study, carried out according to the STROBE (*Strengthening the Reporting of Observational Studies in Epidemiology*) recommendations, using the IntegraSUS database, a Health Transparency platform of the Government of the State of Ceará, about HIV detection rates in pregnant women.

### Sample/Population

The sample consisted of all cases of HIV in pregnant women in the state of Ceará, Northeast Region of Brazil, detected between 2017 and 2021 (n = 1,173), period in which the system presented complete data. In February 2020, the Brazilian Ministry of Health confirmed the first case of the COVID-19 infection, which is considered a landmark of the beginning of the pandemic in Latin America^(16)^.

### Data Collection

The database was accessed in January 2022. All available and analyzed variables were: HIV detection rate in pregnant women, age group, level of education in years of study, proportion of cases according to the time of laboratory diagnosis of HIV infection (before prenatal care, during prenatal care, at delivery or postpartum), proportion of cases according to assessment at prenatal care, proportion of cases according to type of delivery, and proportion of use of antiretrovirals during delivery.

The data collection instrument consisted of the dimensions proposed by the Social Ecological Theory. According to this theory, the environment is considered in levels, as when compared to a set of Russian dolls, in a way that one level is supported by the subsequent level. The levels are classified as: microsystem, mesosystem, exosystem, and macrosystem. In the microsystem, the most internal level, there is the individual’s immediate environment, which includes their closest relationships, such as with family, work, the immediate neighborhood, and school. In the mesosystem (second level), there are the relationships with the first level (microsystem). The exosystem (third level) refers to environments that, although the individual is not present, directly affect their development. Finally, the most external level (macrosystem) involves elements related to temporality, such as systems of values and beliefs^(17)^.

The collection instrument was divided into the characterizing variables of each level proposed by the SET used in a study with HIV^(14)^. In the macrosystem, the HIV detection rate in pregnant women in Ceará was verified. The exosystem was characterized by access to and use of antiretroviral drugs at birth. The mesosystem and microsystem were defined by the age range of the pregnant women, level of education in years of study, prenatal care, time of laboratory diagnosis of HIV infection (before prenatal care, during prenatal care, during delivery, or post-natal care), and the type of delivery.

Data were collected by two researchers independently. Each collection generated a data sheet. Subsequently, the worksheets were evaluated by three other researchers, with a double check leading to a single worksheet. When there was a discrepancy in the information, the public bank was consulted for review of the data collected and adjustment of the final spreadsheet.

### Statistical Analysis

The data were organized and analyzed using the software *Microsoft Excel 2019 and R version 4.1.0*., considering absolute and relative frequencies. Viewing a more specific evaluation of the differences before and after the start of the pandemic, regarding HIV cases in pregnant women, crosses were performed between the pre- and post-pandemic periods, with the Micro/Mesosystem variables: Prenatal care, Type of delivery, Time of diagnosis, Age group, and Women’s education.

To prove the associations, the Chi-Square test of independence was applied to check if there is statistically significant dependence between the mentioned variables, and the Odds Ratio (OR) was calculated, with their respective 95% confidence intervals, to quantify the identified association. The significance level adopted was 5%.

**Figure 1.**
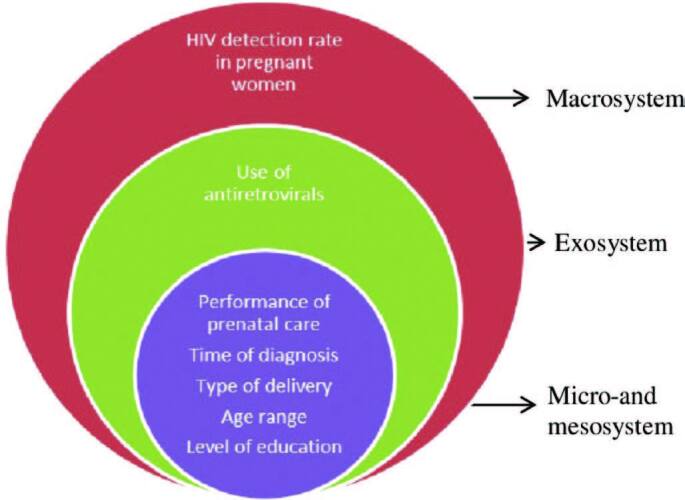
Graphic organization of the variables according to the levels of Social Ecological Theory. Fortaleza, CE, Brazil, 2022.

The analysis was carried out based on the dimensions proposed by the SET: from the most internal (prenatal care, time of diagnosis, type of delivery, age group, and education) to the most external (detection rate), as shown in Figure 1.

### Ethical Aspects

The approval of the Research Ethics Committee was not required to carry out the study, since secondary data from public domain, available via the internet, were used without any identification of the patients.

## RESULTS

In the scenario of the Northeast region of Brazil, focusing on the state of Ceará, 891 cases of HIV were reported in pregnant women from 2017 to 2019, before the onset of the COVID-19 pandemic, and 282 cases between 2020 and 2021, after the onset of pandemic. The detection rate of pregnant women with HIV before the start of the pandemic was 231 women per 100,000 live births, while in the period after the start of the pandemic, this rate dropped to 122.67 women.

Another aspect evaluated in the pre- and post-pandemic period was the exosystem variable on the use of antiretrovirals during childbirth, and the results obtained from this cross are shown in Table 1. It should be noted that 345 records of notified cases (225 before the pandemic and 120 after the start of the pandemic) had this information ignored and, for statistical purposes, these cases were not considered.

**Table 1.** Comparison of the proportions of reported HIV cases in pregnant women before and after the start of the pandemic according to the exosystem level of the Social Ecological Theory – Fortaleza, CE, Brazil, 2022.

Exosystem variable	Period				p value	OR (95%CI)
	Before the start of the pandemic (2017 to 2019) n = 891		After the start of the pandemic (2020 to 2021) n = 282			
	N	%	N	%
Use of antiretrovirals						
Yes	570	63.98	124	43.97	**0.005**	1.00
No	96	10.77	38	13.48		**1.82 (1.19–2.78)**
Ignored*	225	25.25	120	42.55		–

*Category “ignored” was not used in the chi-square test and odds ratio.

Before the COVID-19 pandemic, 63.9% of pregnant women living with HIV used antiretrovirals, and 10.7% did not use them, while after the start of the pandemic, these percentages changed to 43.97% and 13.48% (P < 0.005) respectively.

According to the Odds Ratio (OR), it is possible to state that the use of antiretroviral drugs during childbirth changed after the start of the pandemic, with 1.82 times more chances of pregnant women not using drug therapy during childbirth after the onset of pandemic. Thus, considering that the treatment prevents the vertical transmission of HIV to the child, there is a greater chance of exposure of babies to the virus during the pandemic period.

The data presented in Table 2 refer to the comparisons of micro- and mesosystem variables before and after the start of the pandemic period.

**Table 2.** Association of proportions of reported HIV cases in pregnant women before and after the start of the pandemic according to micro and mesosystem variables of the Social Ecological Theory – Fortaleza, CE, Brazil, 2022.

Micro/mesosystem	Period				p value	OR (95%CI)
	Before the start of the pandemic (2017 to 2019) n = 891		After the start of the pandemic (2020 to 2021) n = 282			
	N	%	N	%
Age range						
Up to 19 years	113	12.69	33	11.7	0.090	1.00
≥20 years	778	87.31	249	88.3		1.27 (0.96–1.68)
Level of education*						
0–9 years of study	406	45.57	139	49.29	0.274	1.16 (0.89–1.52)
>9 years of study	485	54.43	143	50.71		1.00
Prenatal care						
Yes	836	93.83	261	92.55	0.449	1.00
No	55	6.17	21	7.45		1.22 (0.73–2.06)
Time of diagnosis						
Before prenatal care	373	41.86	102	36.17	0.090	1.00
After starting prenatal care	518	58.14	160	63.83		1.27 (0.96–1.68)
Type of delivery						
Vaginal	118	13.24	18	6.38	**<0.001**	**0.45 (0.27–0.75)**
C-section	476	53.43	116	41.14		**0.61 (0.46–0.80)**
Abortion	19	2.13	6	2.13		0.998 (0.39–2.52)
Not informed	278	31.20	142	50.35		**2.24 (1.70–2.94)**

*Years of study.

It was found that after the start of the pandemic, the percentage of cases of vaginal delivery and cesarean section decreased to 6.38% and 41.14%, respectively, while cases of abortion remained the same, and there was an increase in those not informed, to 50.35% after the start of the pandemic (p < 0.001).

However, there was no significant association with regard to prenatal care, time of diagnosis, age group, and education of pregnant women with HIV.

## DISCUSSION

According to the results presented, following data analysis from the perspective of the macrosystem, the COVID-19 pandemic had an impact on the reduction of the HIV detection rate in pregnant women. The decrease in detection may be related to the impact of the pandemic on the overload of health systems, negatively influencing prenatal services, HIV diagnosis, and adequate follow-up, making women more vulnerable to its consequences.

A study carried out in Turkey with the objective of evaluating the accessibility of pregnant women to prenatal screening and diagnostic tests during the pandemic found that there is a notable decrease in the number of prenatal diagnoses and screening tests during the COVID-19 pandemic^(18)^.

To overcome the screening deficiency during prenatal care in the pandemic period, a North American study proposed consolidation of prenatal screening, surveillance, and in-person exams, and fewer in-person visits, maintaining patient access to continuous prenatal care and subspecialty consultations through virtual telemedicine visits^(19)^.

Regarding the exosystem, a significant negative impact of the pandemic on the use of antiretrovirals was observed, since the chances of the pregnant woman not being on treatment increased by 82%. Adherence to antiretroviral treatment during pregnancy, and consequent reduction in viral load, is the most relevant measure for preventing infection in children. However, this therapeutic adherence has proved to be a task difficult to be achieved, including in the pregnancy cycle^(20)^.

Social distancing, which is one of the preventive measures that aims to reduce the transmission of COVID-19, can also lead to serious repercussions and obstacles to prescriptions and receipt of antiretroviral therapy. The challenges become even greater in a context of great social inequality, with populations living in precarious conditions, without sanitation and in an overcrowded situation^(21)^.

The interruption of antiretroviral therapy has a negative impact on HIV control and on the advances that have been made over the last four decades. It is worth noting that the COVID-19 pandemic directly interfered with the treatment and provision of services for people living with HIV. Consultations were rescheduled or routine care hours were reduced, and medication distribution was decreased^(21)^.

Protecting pregnant and lactating women and children from acquiring SARS-CoV-2 while maintaining essential HIV services is an immense global health challenge. Program adaptations, drug delivery, and viral load monitoring for these populations have the potential to limit the transmission of SARS-CoV-2, ensuring continuity of life-saving HIV case identification and treatment efforts^(22)^.

From the SET perspective, the systems closest to the pregnant woman (micro and mesosystem), related to individual characteristics (age, schooling, income) and family relationships, as well as their interactions with their environment, can directly interfere with the development and maintenance of bonds with health services. A qualitative study carried out in Uganda with 47 clients living with HIV and eight HIV service employees, with the objective of identifying the facilitating determinants and barriers to care, identified that users were motivated to attend the HIV outpatient clinic due to the perception of quality services and the belief that antiretroviral therapy improves health. Barriers to clinical care included distance from home, cost of treatment, unemployment, and climate^(23)^.

In the present study, with regard to the micro and mesosystem variables, it was possible to observe that the pandemic had no impact on most of the researched variables. No evidence was found that the age group and education of pregnant women with HIV had changed after the start of the pandemic.

Regarding prenatal care, there was a small proportional decrease in the total number of pregnant women with HIV, before and after the start of the pandemic, but there were no differences in the number of pregnant women with HIV who underwent prenatal care and the chances of having prenatal care were the same before and after the start of the pandemic. It should be noted that prenatal care is of paramount importance to women’s health throughout the pregnancy and childbirth cycle and is associated with better perinatal outcomes. However, the research shows that in the pandemic context, pregnant women have faced difficulties in carrying out and monitoring this assistance, due to the cancellation of consultations, telemedicine consultations, or postponement due to cases of suspected or confirmed COVID-19 infection^(24)^.

Such bonds formed between health care services and the mother during prenatal care are essential to avoid negative outcomes and complications during childbirth. These bonds favor the promotion of qualified care, with early diagnosis and treatment of infections, given the recognition of women’s complaints, in addition to the assessment of referral to high-risk prenatal care, with an active search in cases of interruption of follow-up, as well as the commitment of health professionals to the mother from the whole prenatal period to the puerperium^(25)^.

The time of HIV diagnosis was also not impacted by the pandemic, as the data did not show sufficient evidence that there is a difference when comparing data before and after the pandemic.

When investigating the type of delivery, it was observed that after the start of the pandemic, there was a decrease in the number of vaginal deliveries in women with HIV. There was significant statistic association between the type of delivery and the periods investigated (p < 0.001). This association can result from failure to carry out the tests, reduced quality of obstetric care, which reduced case notification.

It was also found that unreported cases of HIV in pregnant women increased after the pandemic. It was also observed that during this period there are 2.24 times more chances of uninformed cases of pregnant women with HIV.

A systematic review that aimed to analyze the empirical evidence of the indirect impacts of respiratory epidemics on sexual and reproductive health found that COVID-19 resulted in interruptions in services that affected access to contraceptives, tests for HIV and Sexually Transmitted Infections (STIs), and changes in sexual behaviors, menstruation, pregnancy intentions, and increased miscarriages^(26)^.

A Brazilian study that synthesized scientific evidence on gender and racial inequalities in the COVID-19 pandemic, focusing on women’s productive/reproductive work, gender violence, and access to Sexual and Reproductive Health Services confirmed that social inequalities must be considered for the effective control of the pandemic and for the preservation of rights. In addition to the direct effects of COVID-19, it was found that barriers to accessing sexual and reproductive health services can lead to an increase in unintended pregnancies, unsafe abortions, and maternal mortality^(27)^.

During the pandemic, the full performance of women’s care networks to meet their sexual and reproductive rights must be preserved. Immediate care for women in the prenatal period, STI/HIV screening, contraception, adequate follow-up of women living with HIV, access to laboratory tests and quality care is essential.

Thus, evaluating the evolution of HIV cases in pregnant women from before to after the pandemic based on the Social Ecological Theory allowed identifying changes associated with the exosystem and mesosystem. The use of the theory favored a broader analysis of the different factors influencing this health problem.

This study has limitations, such as unrecorded data and/or information not explored in the data system, given the use of secondary data, since they are conditioned to the quality of the records. However, the database used, even with its limitations, is considered to produce reliable information. Information filled in and made available properly can contribute to the quality of care, and is the object of teaching and research in health.

Another finding was the scarce production/disclosure of research involving the theme of HIV in pregnant women in recent years, reflecting in an inversely proportional way to the increase in the number of HIV cases among pregnant women. Moreover, gestational complications caused by COVID-19 are still being clarified, as new cases and studies emerge. Therefore, the great care challenges and gaps in knowledge present in the pandemic scenario are perceived.

## CONCLUSIONS

It is concluded that the COVID-19 pandemic had an epidemiological and care impact on pregnant women living with HIV in the state of Ceará, leading to a reduction in the number of notifications and in the detection rate. In addition, there was great loss in the use of antiretroviral therapy, as well as a decrease in vaginal and cesarean deliveries, and an increase in “unreported” cases after the onset of the COVID-19 pandemic.

The strengthening of the relationship between pregnant women and health professionals, as well as with the community in general, is recommended, so that there is an awareness of the importance of early diagnosis of HIV and effective treatment, with the aim of minimizing vertical transmission and helping manage the infection. The importance of maintaining care for women in the pregnancy cycle in times of COVID-19 shall be emphasized, with the creation of strategies that contribute to ensuring adequate and safe assistance during pregnancy being required.
